# Immune modulation by mesenchymal stem cells

**DOI:** 10.1111/cpr.12712

**Published:** 2019-11-15

**Authors:** Wei Jiang, Jianyong Xu

**Affiliations:** ^1^ Guangdong Provincial Key Laboratory of Regional Immunity and Diseases Health Science Center Shenzhen University Shenzhen China; ^2^ Department of Anatomy, Histology & Developmental Biology Health Science Center Shenzhen University Shenzhen China; ^3^ Department of Immunology Health Science Center Shenzhen University Shenzhen China

**Keywords:** immune modulation, immune modulators, mesenchymal stem cell, stem cell therapy

## Abstract

Mesenchymal stem cells (MSCs) can be derived from various adult tissues with multipotent and self‐renewal abilities. The characteristics of presenting no major ethical concerns, having low immunogenicity and possessing immune modulation functions make MSCs promising candidates for stem cell therapies. MSCs could promote inflammation when the immune system is underactivated and restrain inflammation when the immune system is overactivated to avoid self‐overattack. These cells express many immune suppressors to switch them from a pro‐inflammatory phenotype to an anti‐inflammatory phenotype, resulting in immune effector cell suppression and immune suppressor cell activation. We would discuss the mechanisms governing the immune modulation function of these cells in this review, especially the immune‐suppressive effects of MSCs.

## 1 INTRODUCTION

Mesenchymal stem cells (MSCs), also known as mesenchymal stromal cells, are spindle‐shaped cells with multipotent (chondrocyte, osteoblast and adipocyte) and self‐renewal abilities.^1^, ^2^ These cells are derived from various adult tissues,^3^, ^4^ attach to tissue culture dishes and express certain cell surface markers (positive for CD73, CD90 and CD105; negative for CD45, CD34, CD14 or CD11b, CD79alpha or CD19, and HLA‐DR).^2^ MSCs can be safely harvested with no major ethical concerns and have low immunogenicity.^3^ Therefore, MSCs have been proposed as effective and safe cell sources for stem cell therapy.

Although MSCs have differentiation abilities, the main mechanism of their therapeutic effects in pre‐clinical and clinical studies is believed to be paracrine effects. These paracrine effects include promoting angiogenesis, preventing apoptosis, suppressing inflammation and modulating extracellular matrix dynamics. One of the ways that these cells improve the tissue microenvironments is by modulating immune system components, such as macrophages and neutrophils. After the tissues or cells are injured, the MSCs activate or suppress the immune system to control the whole‐tissue regeneration process.^3^, ^4^, ^5^, ^6^

Mesenchymal stem cells have been successfully applied in treating various diseases such as diabetes,^7^ cardiovascular diseases,^8^ graft‐versus‐host diseases 9 and autoimmune diseases.^10^ Although many questions remain unanswered the immune modulation effects of MSCs make them promising candidates for cell therapy–based tissue repair and disease treatment, especially for immune system abnormalities, such as cancer and autoimmune diseases. Thus, we will discuss the mechanisms of immune modulation by MSCs. Given the important roles of MSCs in immune suppression to help cancer to escape immune surveillance and their potential roles in immune tolerance re‐establishment, we mainly focus on the immune‐suppressive function of MSCs in the current review.

## 2 IMMUNE MODULATION BY MSCS

**Figure 1:**
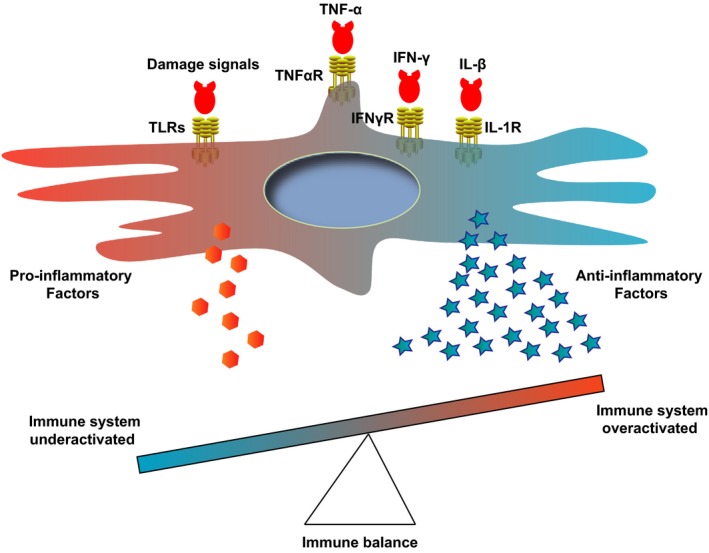
Sensor and switcher model of MSCs. MSCs could sense danger signals through different receptors (such as TLRs) and also respond to excessive pro‐inflammatory signals via receptors for TNF‐α, IFN‐γ and IL‐1β. Depending on the signal types or strength, MSCs secrete cytokines to promote or suppress the immune responses for maintaining the immune balance. IFN‐γ, interferon gamma; IFNγR, interferon gamma receptor; IL‐1R, interleukin‐1 receptor; IL‐1β, interleukin‐1 beta; TLR, Toll‐like receptor; TNF‐α, tumour necrosis factor alpha; TNFαR, tumour necrosis factor alpha receptor

Mesenchymal stem cells could promote inflammation when the immune system is underactivated and restrain inflammation while the immune system is overactivated to avoid self‐overattack. This activity is also known as the function of “sensor and switcher of the immune system” (Figure 1).^11^ The MSCs could sense different danger signals through TLRs (Toll‐like receptors).^12^, ^13^, ^14^, ^15^, ^16^ MSCs express TLR2, TLR3, TLR4, TLR7 and TLR9. The expression levels of these TLRs vary significantly based on their tissue origin.^17^ TLRs recognize molecules from injured cells or pathogens acting as the first line of the immune defence system. TLR activation can further stimulate immune cells and MSCs.^17^ Activated MSCs respond to TLR ligands and release anti‐inflammatory factors. Thus, TLRs play an important role in sensing and switching immune responses by MSCs.^17^ The allogeneic MSCs would be eliminated by NK cells slowly. However, once the MSCs are activated via TLR3 ligand, they could escape from this clearance process by NK cells.^18^ The type of TLR (TLR3 or TLR4) activation could also induce a pro‐inflammatory or anti‐inflammatory phenotype of MSCs.^12^, ^13^, ^14^ For example, TLR3 activation induces an anti‐inflammatory phenotype of MSCs (also known as the MSC2 phenotype), while TLR4 activation induces a pro‐inflammatory phenotype (also known as the MSC1 phenotype).^3^, ^14^

Furthermore, the MSC microenvironment could switch the MSCs between pro‐inflammatory and anti‐inflammatory phenotypes. MSCs have pro‐inflammatory functions in the early stage of inflammation through recruiting neutrophils.^19^ Pro‐inflammatory MSCs activate T cells by secreting MIP‐1 (macrophage inflammatory protein‐1), CCL5 (C‐C motif ligand 5), CXCL9 (C‐X‐C motif ligand 9) and CXCL10 (C‐X‐C motif ligand 10) and recruiting more lymphocytes.^3^ At this stage, there are only low levels of inflammation signals, such as TNF‐α and IFN‐λ. MSCs derived from bone marrow and umbilical cord promote immune response when they are treated with low levels of IFN‐γ and TNF‐α, which could not produce sufficient iNOS or IDO to suppress the lymphocytes.^20^ However, when these two cytokines reach a high level, they stimulate MSCs to secrete iNOS (mice) or IDO (human), resulting in T‐cell proliferation inhibition and Treg induction. Therefore, the iNOS or IDO level has been proposed as the switcher between the pro‐ and anti‐inflammatory effects of MSCs.^3^ TNF‐α and IFN‐λ are often used for MSC activation.^21^

## 3 IMMUNE SUPPRESSION BY MSCS

Immune system components, such as immune molecules and immune cells, protect the host against exogenous pathogen invasion and endogenous cancer development. The understimulated immune system could not protect the host. However, overstimulation would attack the healthy cells and tissues of the host, resulting in tissue or organ destruction. Thus, the immune response must be tightly regulated through different pathways. Uncovering the detailed mechanisms of this regulatory network is critical for understanding the pathogenesis of immune dysfunction–related diseases and developing new therapy strategies. Several cell populations have been demonstrated to prevent immune system overstimulation, including natural and induced CD4^+^ Treg (T regulatory cells),^22^ CD8^+^ Treg,^23^ Breg (B regulatory cells),^24^ M2 macrophages^25^ and suppressive dendritic cells.^26^ These cells modulate the immune reaction through secreting suppressive cytokines, such as IL‐10, TGF‐β, IL‐35, inhibitory ligand and receptors (such as PD‐1 and PD‐L1), and by directly regulating immune cell differentiation, maturation and survival.

It has been demonstrated that MSCs also represent one type of cell to prevent overstimulation of the immune system. The immune‐suppressive activities of MSCs are primarily stimulated by pro‐inflammatory factors, such as IFN‐γ (interferon gamma), TNF‐α (tumour necrosis factor alpha) and IL‐1β (interleukin‐1 beta).^4^, ^5^, ^6^ Among these factors, IFN‐γ is even more crucial for the immune‐suppressive function of MSCs.^27^ IFN‐γ stimulates MSCs to express the immune inhibitors PD‐L1 and PD‐L2 (programmed cell death ligands 1 and 2) and downregulates ILTRs (immunoglobulin‐like transcript receptors).^28^

The immunosuppressive MSCs have downregulated antigen‐presenting molecules (MHC‐I, MHC‐II), co‐stimulators (CD80, CD86, CD40, CD40L) and FasL.^3^, ^21^ MSCs also express many chemokines and adhesion proteins to recruit immune cells, such as CXCR3 (C‐X‐C motif chemokine receptor 3) ligands, CCR5 (C‐C motif chemokine receptor 5) ligands, ICAM‐1 (intercellular adhesion molecule 1) and VCAM‐1 (vascular cell adhesion molecule 1).^3^, ^21^ MSCs could suppress the inflammation process, partly through downregulating pro‐inflammatory factors and upregulating anti‐inflammatory factors. Furthermore, these cells could suppress immune reactions through direct cell contact.

### 3.1 Immune modulators expressed by MSCs

**Figure 2:**
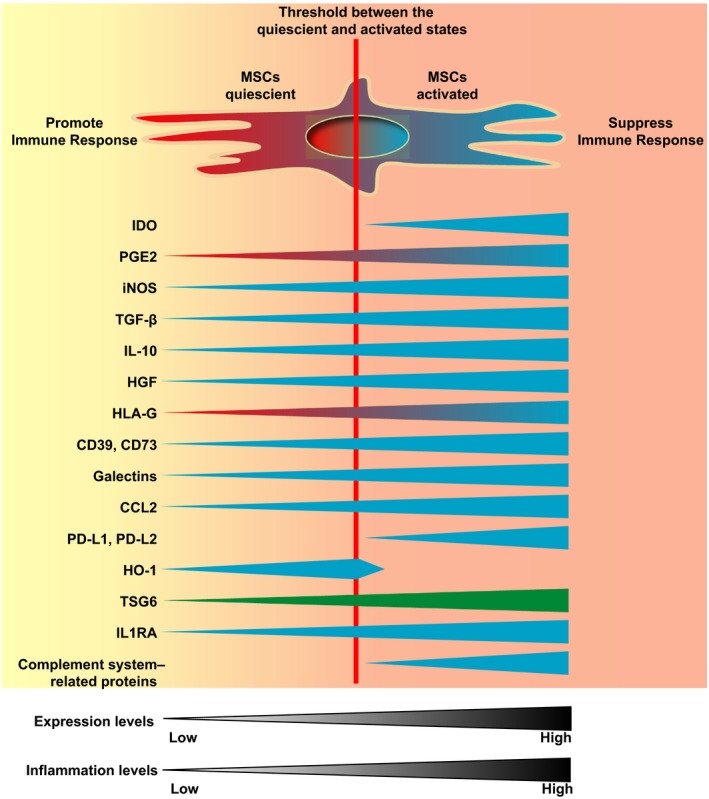
Immune modulators expressed by MSCs. MSCs express many immune modulators under different conditions. Some modulators are expressed in both quiescent and activated states, including PGE2, iNOS, TGF‐β, IL‐10, HGF, CD39 and CD73, galectins, CCL2, TSG6 and IL1RA. Some are expressed only in the activated state, including IDO, PD‐L1 and PD‐L2, and complement system–related proteins. And the HO‐1 is mainly expressed in the quiescent state and decreased sharply in the activated state of MSCs. However, they all are upregulated by pro‐inflammatory factors in the concentration‐dependent manner. Data have shown that low levels of PGE2 and HLA‐G have pro‐inflammation effects, while high levels have anti‐inflammation effects. TSG6 is mainly expressed in the MSC sphere state. Blue and green indicate the anti‐inflammation function; red indicates the pro‐inflammation function. CCL2, C‐C motif chemokine ligand 2; HGF, hepatocyte growth factor; HLA‐G, histocompatibility locus antigen‐G; HO‐1, haem oxygenase 1; IDO, indoleamine 2,3‐dioxygenase; IL‐10, interleukin‐10; IL1RA, interleukin‐1 receptor antagonist; iNOS, inducible nitric oxide synthase; MSCs, mesenchymal stem cells; PD‐L1 and PD‐L2, programmed cell death ligands 1 and 2; PGE2, prostaglandin E2; TGF‐β, transforming growth factor beta; TSG6, tumour necrosis factor‐stimulated gene 6

Although direct cell contact is important for the immune‐suppressive effects of MSCs, studies have shown that the immune modulators expressed by MSCs are more critical, including indoleamine 2,3‐dioxygenase (IDO), prostaglandin E2 (PGE2), inducible nitric oxide synthase (iNOS), transforming growth factor beta (TGF‐β), interleukin‐10 (IL‐10), hepatocyte growth factor (HGF), histocompatibility locus antigen‐G (HLA‐G), CD39 and CD73, galectins, C‐C motif chemokine ligand 2 (CCL2), programmed cell death ligands 1 and 2 (PD‐L1 and PD‐L2), haem oxygenase 1 (HO‐1), tumour necrosis factor‐stimulated gene 6 (TSG6), interleukin‐1 receptor antagonist (IL1RA) and complement system–related factors (Figure 2).

#### 3.1.1 IDO

IDO has two isoforms, IDO1 and IDO2. These isoforms catalyse tryptophan, an important essential amino acid, into different metabolites, resulting in tryptophan depletion.^29^ Because tryptophan is essential for T‐cell proliferation,^30^ tryptophan depletion switches the metabolic pathway from glycolysis to oxidative phosphorylation, resulting in T‐cell arrest.^31^ Tryptophan reduction also induces the accumulation of uncharged tryptophan tRNA in immune cells, which could activate stress‐response kinase GCN2 (general control nonderepressible 2) and eIF2 (eukaryotic translation initiation factor 2)‐mediated pathways, leading to protein synthesis reduction, cell proliferation inhibition and Fas‐mediated lymphocyte apoptosis.^32^ GCN2 pathway activation also promotes Treg differentiation while suppressing Th17 conversion through downregulating IL‐6.^33^ Tryptophan deprivation could induce Treg generation through producing tolerogenic DCs, with downregulation of co‐stimulatory molecules and upregulation of the inhibitory receptors ILT3 (immunoglobulin‐like transcript 3) and ILT4 (immunoglobulin‐like transcript 4) on DCs.^34^ The tryptophan metabolites (kynurenine, quinolinic acid and picolinic acid) are more toxic to CD4^+^ Th1 and CD8^+^ T cells and less toxic to Th2 cells, thereby switching T helper cells from Th1 to Th2.^35^ Furthermore, the tryptophan metabolite kynurenine could directly bind to AhR (aryl hydrocarbon receptor) and promote CD4^+^Foxp3^+^ Treg differentiation while suppressing Th17 generation and decreasing DC immunogenicity.^36^

IDO is primarily expressed by antigen‐presenting cells.^32^ MSCs also express and utilize IDO to mediate immune suppression.^37^ IDO is not expressed in MSCs in the quiescent state but could be induced by IFN‐*γ* and enhanced by PGE2. Under IFN‐*γ* stimulation, activated STAT1 (signal transducer and activator of transcription 1), IRF‐1 (interferon regulatory factor‐1) and NF‐B (nuclear factor kappa‐light‐chain‐enhancer of activated B cells) bind to the upstream IFN‐*γ*–responsive elements of the IDO gene and promote IDO gene expression.^38^, ^39^

#### 3.1.2 PGE2

PGE2 is produced by COX‐1 (cyclooxygenase‐1, the constitutive isoform) or COX‐2 (cyclooxygenase‐2, the inducible isoform) from the arachidonic acid released from the membrane phospholipids. PGE2 interacts with EP2 and EP4 receptors expressed on the surface of immune cells and exerts its anti‐inflammatory effects. The interaction between PGE2 and EP2 or EP4 receptors induces cyclic AMP (cAMP) upregulation, which then activates the PKA (protein kinase A) and PI3K (phosphatidylinositol‐3 kinase) pathways. cAMP induces the expression of anti‐inflammatory factors (IL‐4, IL‐5 and IL‐10) and inhibits the expression of pro‐inflammatory factors (IL‐12p70, TNF‐*α*, CCL3 and CCL4) through IL‐2 pathway suppression. In addition, cAMP promoted M2 macrophage and Th2 cell differentiation and inhibited Th1 production.^40^, ^41^, ^42^ However, some studies have shown that PGE2 has pro‐inflammatory effects with enhancing DC maturation and T‐cell proliferation.^43^ Later studies have demonstrated that a low concentration of PGE2 promotes an inflammatory response, while a high concentration inhibits.^43^ PGE2 promotes Foxp3^+^ Treg cell production.^44^ PGE2 also promotes TGF‐*β* secretion from monocytes and induces MDSC (myeloid‐derived suppressor cells) generation, which could suppress NK cell and CD8^+^ T‐cell activities.^45^, ^46^

PGE2 suppresses IL‐12 and promotes IL‐23 expression. IL‐12 (IL‐12p70) is composed of IL‐12p35 and IL‐12p40. The suppression of IL‐12 by PGE2 is mediated through inhibiting IL‐12p35 but not IL‐12p40. PGE2 could increase IL‐23p19 expression, which could form IL‐23 with IL‐12p40. Thus, PGE2 induces IL‐23 expression, which is important for Th17 production.^47^, ^48^

MSCs express COX‐2 and produce PGE2,^11^, ^49^ which could be further enhanced by inflammatory stimuli or the combination of IFN‐*γ* and TNF‐*α* treatment.^50^ Therefore, these cells produce high amounts of PGE2 to suppress the immune response.^51^

#### 3.1.3 iNOS

Mesenchymal stem cells express iNOS, which metabolizes L‐arginine to generate NO (nitric oxide).^37^, ^52^ NO suppresses the IL‐2 pathways (Janus kinase 3, signal transducer and activator of transcription 5, extracellular signal–regulated kinases and protein kinase B), resulting in T‐cell proliferation and function inhibition.^52^, ^53^, ^54^, ^55^ NO also induces T‐cell apoptosis and inhibits the expression of MHC‐II.^56^ NO suppresses the secretion of Th1 and Th2 cytokines.^57^, ^58^ When MSCs are stimulated with inflammatory factors, the iNOS gene is upregulated. These cells produce high amounts of NO to suppress the immune response.^21^, ^51^ Interestingly, the pro‐inflammatory cytokine IL‐17 could stabilize the iNOS protein in MSCs derived from bone marrow, resulting in immune suppression.^59^

MSCs from mice, rabbits, rats and hamsters mainly exert suppressive functions through iNOS, while MSCs derived from humans, pigs and monkeys primarily exert suppressive functions through IDO.^60^ Thus, the mechanism of immune‐suppressive functions of MSCs from different species might differ in the detailed pathways.

#### 3.1.4 TGF‐β

TGF‐β and IL‐10 are the main immune‐regulatory cytokines generated by quiescent MSCs.^61^, ^62^ TGF‐β is constitutively secreted by MSCs 63 and further upregulated by inflammatory factors, such as IFN‐γ and TNF‐α.^50^, ^64^, ^65^ TGF‐β inhibits IL‐2, MHC‐II (major histocompatibility complex II) and co‐stimulatory factor expression in DCs and T cells.^61^, ^62^ Both Th1 differentiation and Th2 differentiation could be inhibited by TGF‐β.^66^, ^67^ TGF‐β promotes Treg and Breg production.^61^ TGF‐β is one of the key regulators of Foxp3 expression.^61^, ^62^ However, it has also been shown that the immune suppression effects of bone marrow‐derived MSCs stimulated with IFN‐γ and TNF‐α are abolished by adding TGF‐β through inhibiting iNOS and IDO expression.^68^

#### 3.1.5 IL‐10

In addition to TGF‐β, IL‐10 is another main immune‐suppressive cytokine generated by quiescent MSCs. IL‐10 expression could be further enhanced by TLR ligands and PEG2.^69^ IL‐10 could inhibit antigen‐presenting cell (APC) maturation and the expression of MHC and co‐stimulatory factors.^70^ IL‐10 inhibits pro‐inflammatory production, T‐cell proliferation and memory T‐cell formation.^70^ IL‐10 suppresses Th17 generation and promotes Treg formation.^71^ IL‐10 exerts its anti‐inflammatory effects through the JAK1‐TYK2‐STAT3‐SOCS3 pathway.^72^

#### 3.1.6 HGF

MSCs express HGF, which exhibits immune suppression effects. HGF induces IL‐10 expression in monocytes, inhibits Th1 and DC activities, and promotes IL‐10–positive Treg cells.^73^, ^74^ HGF generated by MSCs also promotes immune‐suppressive MDSC expansion.^75^

#### 3.1.7 HLA‐G

MSCs secrete HLA‐G5 (one secreted isoform of non‐classical class I MHC with immune‐suppressive functions) under the stimulation of IL‐10, IFN‐γ and TNF‐α.^76^ HLA‐G binds to the receptors of ILT2 and ILT4, which are widely expressed by monocytes/macrophages, DCs, CD4^+^ and CD8^+^ T cells, B cells and NK cells.^77^ HLA‐G inhibits the cytotoxic function of CD8^+^ T and NK cells, cytokine production of Th1 and Th17 cells, and induces Treg generation and MDSC expansion.^76^, ^78^, ^79^ However, the immune‐suppressive effects of HLA‐G might also be concentration‐dependent. It has been shown that a high concentration of HLA‐G induces Treg generation, while a low concentration promotes Th1 development.^80^ HLA‐G also confers the immune privilege characteristics of MSC differentiated derivatives 81, 82

#### 3.1.8 CD39 and CD73

MSCs express CD39 and CD73. CD39 catabolizes ATP to AMP, and CD73 catabolizes AMP to adenosine. Extracellular ATP has pro‐inflammatory effects, while adenosine has anti‐inflammatory effects through the cAMP and PKA pathways. Thus, CD39 and CD73 could cleave extracellular ATP to adenosine and switch pro‐inflammation to anti‐inflammation.^83^, ^84^

#### 3.1.9 Galectins

Galectins (Gal) are soluble proteins that bind to cell surface glycoproteins. MSCs express three isoforms of Gal, Gal‐1, Gal‐3 and Gal‐9.^85^, ^86^, ^87^ Gal‐1 binds to Th1 and Th17 but not Th2 cells and induces cell apoptosis.^88^ Furthermore, Gal‐1 promotes IL‐10 production in Th1 and Th17 cells.^89^ Gal‐1 suppresses the migration of immunogenic DCs.^89^ Gal‐1 and Sema‐3A bind to NRP1 (neuropilin 1, expressed on the T‐cell surface) and arrest the T cells in the G0/G1 phase.^90^ Gal‐9 suppresses B‐ and T‐cell proliferation and is upregulated by IFN‐γ.^91^

#### 3.1.10 CCL2

Mesenchymal stem cells express CCL2 and the related metalloproteinases that are responsible for CCL2 cleavage. The truncated CCL2 functions as a CCR2 antagonist and inhibits immune cell migration. While the full‐length CCL2 binds to its receptor CCR2, which is expressed by activated Th1, Th17 and NK cells, and recruits them into the inflammation sites, the truncated CCL2 plays a critical role in the autoimmunity suppression by MSCs.

#### 3.1.11 PD‐L1 and PD‐L2

MSCs express PD‐L1 (B7H1) and PD‐L2 (B7DC) under IFN‐*γ* and TNF‐α stimulation.^92^, ^93^, ^94^ Blocking the PD‐L1 and PD‐L2 pathways significantly impairs the immune‐suppressive effects of MSCs.^92^, ^93^ MSCs secreted PD‐L1/L2 bind to PD‐1 and inhibit lymphocyte proliferation.^94^, ^95^ PD‐L1 and PD‐L2 could suppress CD4^+^ T‐cell activation, reduce IL‐2 secretion, silence T cells and induce T‐cell death.^94^ These factors could also inhibit AKT phosphorylation and upregulate Foxp3 expression, resulting in Treg production.^94^

#### 3.1.12 HO‐1

Both human and rat MSCs express a high level of HO‐1 in the quiescent state.^96^ Blocking HO‐1 reduced the immune‐suppressive effects of MSCs.^96^ HO‐1 could promote IL10^+^ Tr1 and TGFβ^+^ Tr3 generation, two types of Treg.^97^ However, once MSCs are activated by pro‐inflammatory factors, HO‐1 expression decreases rapidly, and the immune‐suppressive function of MSCs is taken over by other suppressive factors, such as iNOS.^97^

#### 3.1.13 TSG6

The aggregated MSCs and MSC spheres express TSG6, an important immune‐suppressive factor.^98^, ^99^ TSG6 could reduce lymphocyte and neutrophil proliferation and decrease metalloproteinase activity and the expression of IL‐6 and IFN‐γ. On the other hand, TSG6 could promote Foxp3^+^ Treg and IL10^+^iNOS^+^ regulatory macrophage expansion.^98^

#### 3.1.14 IL1RA

IL1RA expressed by MSCs could promote M2 macrophage polarization and Treg generation with elevated IL‐10 expression and suppress CD4^+^ T‐cell activities. Furthermore, IL1RA could suppress B‐cell differentiation and antibody production.^100^, ^101^

#### 3.1.15 Complement system–related proteins

MSCs express C3aR (C3a receptor) and C5aR (C5a receptor), which could be activated by C3a and C5a produced in the inflammation sites. The activated C3aR/C5aR could enhance the resistance to oxidative stress and apoptosis of MSCs.^102^ On the other hand, CD46, CD55 and CD59 expressed on the surface of MSCs could inhibit complement system activation and prevent MSCs from cell lysis.^102^, ^103^ However, once cell lysis is activated by the complementary system, this protection is not sufficient to stop the cell death process.^104^ Combining IFN‐γ treatment with TNF‐α significantly increases the ability of MSCs to secrete factor H, which is a key molecule involved in the inhibition of complement activation.^103^

### 3.2 Immune cells modulated by MSCs

**Figure 3:**
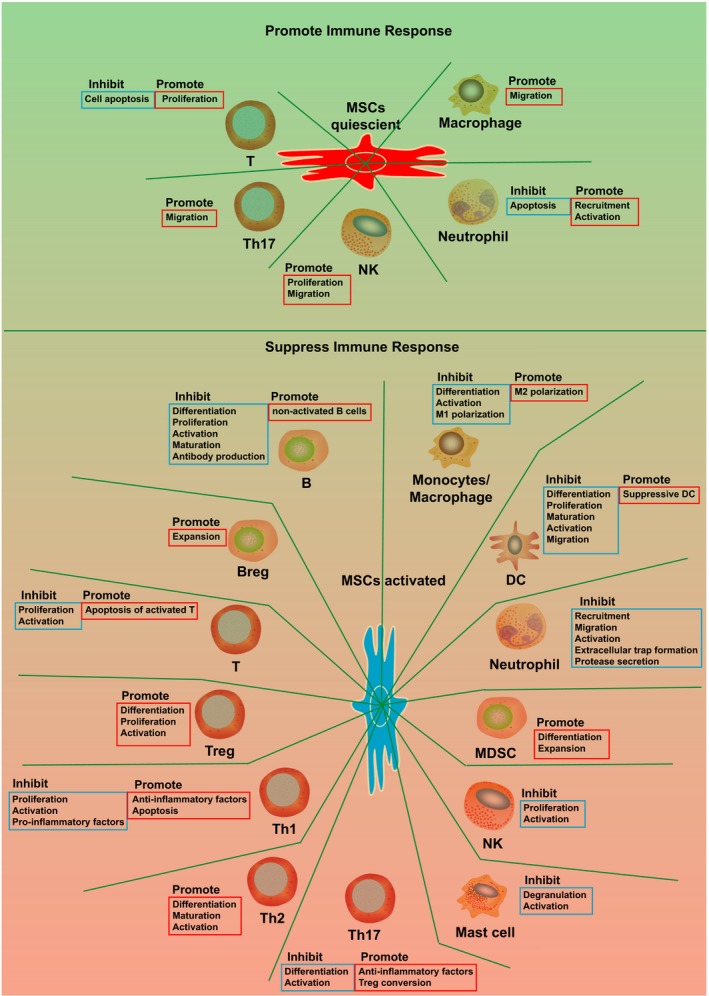
Immune cells modulated by MSCs. MSCs regulate many immune cells from different perspectives, either promoting or suppressing the immune cells. Blue frame indicates inhibiting the functions; red frame indicates promoting the functions. Breg: regulatory B cell; DC: dendritic cell; MDSC: myeloid‐derived suppressor cells; MSCs: mesenchymal stem cells; NK: natural killer cell; Th1: T helper cell, type 1; Th17: T helper cell, type 17; Th2: T helper cell, type 2; Treg: regulatory T cell

MSCs regulate many types of immune cells through the immune modulators expressed by them, including DC (dendritic cell), monocyte/macrophage, B, Breg (regulatory B cell), T, Treg (regulatory T cell), Th1 (T helper cell, type 1), Th2 (T helper cell, type 2), Th17 (T helper cell, type 17), NK (natural killer cell), NKT (natural killer T cell), ILC (innate lymphoid cell), MDSC (myeloid‐derived suppressor cells), neutrophils and mast cells (Figure 3).^62^

#### 3.2.1 DC

Mesenchymal stem cells suppress DC differentiation, maturation and activation and compromise their antigen presentation abilities.^105^, ^106^, ^107^, ^108^, ^109^ MSCs inhibit DC differentiation from monocytes or CD34^+^ HSCs (hematopoietic stem cells), resulting in immature DC production and immune suppression.^105^, ^110^ MSCs could downregulate the expression of HLA II, CD80, CD86 and IL‐12 in DCs, resulting in the inhibition of DC maturation and activation.^110^, ^111^, ^112^

For mature DCs, MSCs could inhibit DC migration by downregulating CCR7 and CD49dβ1 and decreasing inflammatory factor expression and antigen presentation abilities.^109^, ^113^, ^114^, ^115^ MSCs could switch the mature DCs into a suppressive immature phenotype through the Jagged1 or IL‐10‐SOCS3 pathway.^116^, ^117^, ^118^ MSCs promote IL‐10–positive pDCs (plasmacytoid DCs) differentiation,^11^ which would promote Treg development.^119^

Mesenchymal stem cells could also induce DCs into an anti‐inflammatory phenotype through downregulating the pro‐inflammatory factors (TNF‐α and IL‐12) and upregulating the anti‐inflammatory factors, such as IL‐10,^11^, ^120^ PGE2^121^, ^122^ and M‐CSF (macrophage colony–stimulating factor).^123^, ^124^ MSCs induce DCs to secrete IL‐10 and then inhibit T‐cell activation.^11^, ^120^ MSCs also express high levels of PGE2, which binds to its receptor EP4 on DCs and exerts inhibitory effects.^121^, ^122^

The direct contact between MSCs and DCs activates the Notch pathway and suppresses DC generation^106^ and proliferation.^125^ Furthermore, MSCs block the cell interaction between DCs and lymphocytes.^126^ On the other hand, DCs support MSC survival through lymphotoxin‐β expression.^127^

#### 3.2.2 Monocytes/Macrophages

Monocyte modulation is the critical step in the immune modulation process, as depleting monocytes would abolish the immune‐suppressive effects of MSCs.^128^, ^129^ These results support the hypothesis that the immune‐suppressive effects of MSCs are mainly induced through monocyte/macrophage modulation by MSCs.

Mesenchymal stem cells inhibit monocyte differentiation from CD34^+^ HSCs.^105^, ^122^, ^123^ These cells could also induce M2 macrophage polarization, which expresses high levels of immune‐suppressive factors (such as IL‐10) and low levels of immune activators (such as IL‐6, IL‐12, TNF‐α, IL‐1β, IL‐23, CD86 and MHC‐II).^108^, ^128^, ^129^, ^130^, ^131^, ^132^, ^133^ MSCs derived from bone marrow and placenta could induce tolerogenic monocytes and M2 macrophages through IL‐10 and B7‐H4 expression.^108^, ^134^, ^135^ The TGF‐β pathway is also involved in the M2 macrophage polarization process mediated by MSCs.^136^

Under an inflammatory environment, MSCs could recruit macrophages to inflamed sites and enhance tissue regeneration and immune regulation.^137^, ^138^ MSCs sensitize inflammatory factors and switch macrophages from the M1 (pro‐inflammatory) to the M2 (anti‐inflammatory) phenotype through IDO (indoleamine 2,3‐dioxygenase), CCL18 (C‐C motif ligand 18) and PGE2 (prostaglandin E2).^63^ The M2 macrophage polarization effect of MSCs is further enhanced by pro‐inflammatory factor stimulation.^101^, ^128^, ^134^, ^139^, ^140^, ^141^, ^142^, ^143^ Inflammatory factors (such as IFN‐γ, TNF‐α and LPS) could stimulate MSCs to express immune‐suppressive factors, such as PGE2, IDO and COX2.^42^, ^143^ The PGE2 released by MSCs binds to its receptors EP2 and EP4 on macrophages and activates downstream pathways to polarize macrophages to M2 phenotype.^134^ MSCs also express IL‐1RA to suppress the immune response and induce M2 macrophage polarization.^101^, ^140^

Furthermore, the activated MSCs secrete the immune‐suppressive factor TSG‐6 and inhibit the activation of newly differentiated macrophages.^139^ TSG‐6 interacts with CD44 expressed on macrophages and decreases the nuclear translocation of NF‐κB.^139^

#### 3.2.3 B cells

Although the detailed mechanisms are still lacking, it has been demonstrated that MSCs inhibit B‐cell differentiation, proliferation, activation and antibody production indirectly or directly.^101^, ^144^, ^145^, ^146^, ^147^, ^148^, ^149^, ^150^, ^151^, ^152^, ^153^, ^154^, ^155^

MSCs secrete IL‐1RA to inhibit B‐cell maturation.^101^ MSCs secrete CCL2, which inhibits STAT3 activation and induces PAX5 expression, to suppress antibody production in B cells.^150^ MSCs also directly inhibit B‐cell activity through the PD‐1/PD‐L1 pathway.^156^

Furthermore, MSCs promote non‐activated B cells (naive, transitional and memory subsets) formation.^148^, ^150^, ^157^ Non‐activated B cells promote Treg differentiation.^158^ However, it was also shown that MSCs could induce purified B‐cell proliferation and differentiation.^157^ Later studies have demonstrated that B‐cell suppression by MSCs requires signals from T cells.^147^

#### 3.2.4 Regulatory B cells

Mesenchymal stem cells could promote Breg production (CD19^+^CD24^high^CD38^high^ in humans and CD19^+^CD1d^high^CD5^+^ in mice) with IL‐10 expression.^9^, ^101^, ^148^, ^154^, ^159^, ^160^ The expansion of Breg cells promoted by MSCs might account for the total B‐cell population expansion in some studies.^152^, ^161^

#### 3.2.5 T cells

Mesenchymal stem cells inhibit T‐cell proliferation and activation regardless of the species and tissue origins.^162^, ^163^, ^164^, ^165^, ^166^, ^167^, ^168^, ^169^, ^170^, ^171^ MSCs inhibit T cells directly or indirectly by inducing the suppressive Tr1 (CD4^+^IL‑10^+^ cells) 172 and Treg cells (CD4^+^CD25^+^Foxp3^+^ or CD4^+^IL‐10^+^IFN‐γ^+^),^6^, ^173^ which would further inhibit the T cells.^174^, ^175^ MSCs express erythropoietin‐producing hepatocellular (EPH) receptor B2 (EPHB2) and ephrinB2, while T cells express EPHB4 and ephrinB1.^163^ The direct interaction between MSCs and T cells through EPHB2/ephrinB1 and ephrinB2/EPHB4 is essential for the immune‐suppressive effects, as blocking these interactions reduces the suppressive effects.^163^ Furthermore, these interactions would enhance the expression of IDO and iNOS.^163^ EPHB2 and ephrinB2 treatment decreased the expression of TNF‐α, IL‐2 and IL‐17 in T cells.^176^

The T‐cell suppression effects of MSCs rely on the high cell ratio of MSCs to T cells.^166^ The low cell ratio stimulates T‐cell proliferation.^162^ MSCs even protect T cells from apoptosis in the quiescent state.^177^ Activated MSCs express PD‐L1 and FasL, which inhibit CD69 expression and T‐cell proliferation.^178^ MSCs also express HLA‐G1, TGF‐β and HGF to inhibit T‐cell proliferation through downregulating phosphoretinoblastoma (pRb), cyclin D and cyclin A while upregulating cyclin‐dependent kinase inhibitor 1B (p27Kip1), resulting in cycle arrest to G1 phase.^168^, ^169^, ^170^, ^179^ MSCs could induce activated T cells into apoptosis through converting tryptophan into kynurenine 180 and the Fas/FasL pathway.^181^ MSCs secrete galectin‐1, galectin‐3 and galectin‐9 to inhibit T‐cell activities.^85^, ^86^, ^87^ The interactions between galectin‐9 and TIM3 (T‐cell immunoglobulin domain and mucin domain 3) lead to cell apoptosis.^91^, ^182^, ^183^ Furthermore, MSCs inhibit the antigen‐specific proliferation of memory T cells 166 and induce memory Treg cells (CD3^+^CD45RO^+^).^184^ However, the T‐cell suppression effects of MSCs could be abolished by treatment with IL‐2.^168^

#### 3.2.6 Regulatory T cells

Mesenchymal stem cells promote Treg differentiation through both direct cell contact and paracrine effects (such as PGE2, TGF‐β, HLA‐G5 and IL‐10).^76^, ^185^, ^186^ MSCs could directly induce Treg differentiation through the TLR‐Notch pathway^118^, ^187^, ^188^ and the secretion of TGF‐β1 (transforming growth factor beta 1), IDO and iNOS (inducible nitric oxide synthase).^63^ MSCs also promote IL‐10 production and inhibit IFN‐γ and IL‐17 secretion, resulting in promoting Treg differentiation of CD4^+^ T cells and suppressing Th1 and Th17 differentiation.^76^, ^189^ Furthermore, MSCs express GILZ (glucocorticoid‐induced leucine zipper) to induce regulatory Th17 cells with immune‐suppressive effects^190^ and Treg cells.^191^ MSCs promote CD8^+^CD28^‐^ Treg generation and activities^192^ through upregulating IL‐10 and FasL.^192^ MSCs also promote IL10^+^ Tr1 and TGFβ^+^ Th3 production through HO‐1.^97^

#### 3.2.7 Th1

Mesenchymal stem cells exert immune‐suppressive effects through inhibiting Th1 type pro‐inflammatory factor expression (such as IFN‐γ, TNF‐α and IL‐1β) and enhancing Th2 type factor expression.^11^ MSCs promote Th1 cells to secrete the immune suppressor IL‐10 and thus repress the immune responses.^193^ MSCs also inhibit Th1 cell activation indirectly through suppressing DC and NK cells.^113^

#### 3.2.8 Th2

Mesenchymal stem cells induce the differentiation and maturation of Th2 cells through IDO expression, which causes tryptophan depletion and tryptophan metabolite production.^194^ The tryptophan metabolites also induced Th1 cell apoptosis.^195^

#### 3.2.9 Th17

Mesenchymal stem cells could inhibit Th17 cell differentiation and function directly or indirectly.^196^ MSCs enhance CD54 expression and recruit Th17 cells onto MSCs through CCR6‐CCL20.^186^ MSCs could inhibit Th17 differentiation through upregulating PD‐1, IL‐10, CCL2 or SOCS3^186^, ^197^, ^198^, ^199^ and inhibiting the STAT3 pathway.^198^, ^199^ STAT3 pathway inhibition reduces Th17 differentiation through downregulating RORt and IL‐17 expression.^200^ Th17 inhibition by MSCs also involves PGE2.^186^ The MSCs could even convert the Th17 cells into Treg cells.^201^ Although some studies have shown that MSCs could promote Th17 expansion,^202^, ^203^ these studies have limitations that might require further investigation. For example, Guo et  al^202^ demonstrated that bone marrow‐derived MSCs promoted IL‐17 expression and Th17 cell differentiation in mixed lymphocyte reaction experiments. However, these researchers did not find the upregulation of Treg cell,^202^ which should be normally observed in this assay. Thus, the quality and quantity of the MSCs might affect the findings.

#### 3.2.10 NK

MSCs could inhibit the proliferation, activation and activities of NK cells.^11^, ^204^, ^205^, ^206^, ^207^, ^208^, ^209^ However, the inhibitory effects of MSCs on NK cells also require a high cell ratio of MSCs to NK cells.^205^ IDO and PGE2 play important roles in these suppressive effects.^205^, ^206^ MSCs induce NK cells to upregulate CD73 expression.^210^ CD73 could convert AMP into adenosine, the anti‐inflammation inducer.^211^ The TLR4 expressed on MSCs also mediates direct contact with NK cells.^208^, ^212^ Furthermore, MSCs inhibit the activation and proliferation of γδT cells and invariant NKT cells.^213^

On the other hand, it has been demonstrated that MSCs support NK cell proliferation at the low cell ratio of MSCs to NK cells.^214^ IL‐12‐ or IL‐18–stimulated MSCs promote IFN‐γ secretion from NK cells.^215^ Furthermore, the NK cell–secreted IFN‐γ promotes MSCs expressing CCL2, which would further enhance IFN‐γ secretion from NK cells.^216^ NK cells also secrete CCL5 and CXCL7 to recruit MSCs.^217^ Activated NK cells could induce MSC death.^205^, ^218^

#### 3.2.11 NKT

MSCs inhibit the expansion and activity of NKT cells through both direct cell contact and paracrine modulators,^219^, ^220^ such as PGE2,^221^ IDO^222^ and iNOS.^223^

#### 3.2.12 ILC

Mesenchymal stem cells support the differentiation of ILC2 (group 2 innate lymphoid cells),^224^ and the expansion and activity of ILC3 (group 3 innate lymphoid cells).^225^ Furthermore, ILC3 also promotes the activity of MSCs.^225^ Both ILC2 and ILC3 have tissue‐protective functions, such as anti‐inflammation and promoting tissue regeneration.^226^, ^227^ Recently, it has been demonstrated that ILC3 could support and generate Treg cells by secreting IL‐2.^228^

#### 3.2.13 MDSC

MSCs promote MDSC (CD14^‐^CD11b^+^CD33^+^ in humans and Gr‐1^+^CD11b^+^ in mice) generation and expansion.^75^ MSCs could induce MDSCs to express iNOS and arginase, which suppress T‐cell activity and promote Treg expansion. These supportive effects on MDSCs occur through HGF secretion from MSCs. HGF interacts with its receptor c‐Met expressed by MDSCs, induces phosphorylation of STAT3 and thus promotes MDSC proliferation.^75^

#### 3.2.14 Neutrophils

MSCs suppress neutrophil recruitment, activation, extracellular trap formation and protease secretion by secreting superoxide dismutase‐3.^229^, ^230^, ^231^ However, some reports have shown that MSCs protect neutrophils from apoptosis, promote their function through the IL‐6 and STAT3 pathways,^232^, ^233^, ^234^ and promote neutrophil recruitment through IL‐8 and MIF (macrophage migration inhibitory factor) secreted by MSCs.^235^ This feature is correlated with the pro‐inflammation phenotype of MSCs.^12^, ^107^, ^236^, ^237^ Thus, neutrophil modulation by MSCs might also depend on the pro‐inflammatory or anti‐inflammatory phenotype of MSCs.

#### 3.2.15 Mast cells

Mesenchymal stem cells could inhibit the immune activities of mast cells, including inflammatory cytokine expression, degranulation and chemotaxis abilities, via COX2‐PGE2 and TGF‐β1 pathways.^238^, ^239^, ^240^, ^241^, ^242^ MSCs secrete PGE2 via upregulating COX2. Then, PGE2 recognizes and activates the EP4 receptor expressed on mast cells, resulting in mast cell suppression.^240^

## 4 CONCLUSION

Although further efforts should be made to understand the biological roles of MSCs in immunological modulation, the basic concept about the function of MSCs is becoming clear. Perivascular MSCs sense danger signals through receptors, such as TLRs. Then, the MSCs recruit immune cells and promote inflammation. At the later stage of inflammation, MSCs are activated by excessive pro‐inflammatory factors and begin to suppress inflammation to avoid self‐attack. These cells express many immune suppressors to switch them from a pro‐inflammatory phenotype to an anti‐inflammatory phenotype, resulting in immune effector cell suppression and immune suppressor cell activation.

## CONFLICT OF INTEREST

The authors declare no commercial or financial conflict of interest.

## AUTHOR CONTRIBUTION

WJ collected the updated references; JX wrote the manuscript and draw the figures.

## FUTURE PERSPECTIVES

Since the first demonstration of MSCs, many achievements have been made to understand their localization, function and underlying mechanisms. However, many unresolved issues still need to be addressed. In this section, we would like to raise three main questions that should be answered in the coming future. First, what is the specific cell marker for MSCs? Several MSC markers have been demonstrated, but none of them are specific.^243^ Second, what is the role of MSCs in situ? Most of the studies to date are based on in vitro experiments or transplanting the in vitro expanded MSCs into the host. Whether or how much the in vitro culture conditions could affect the phenotype or function of MSCs requires further investigation. Third, is it the time for us to reconsider the appropriateness of the MSC definition? It has been understood for a long time that MSCs are heterogeneous. The MSCs defined by current widely used criteria actually contain several cell populations. Heterogeneity may make the research conclusions controversial. Instead of studying the mixed populations of MSCs, in the future, it might be better to focus on specific subpopulations with specific cell markers and functions.
